# Reactions and Feelings to a Close Relative’s Coming Out in a Heterosexual Relationship

**DOI:** 10.3389/fpsyg.2022.836428

**Published:** 2022-05-06

**Authors:** Claudia Niedlich, Melanie C. Steffens, Janine Dieckmann

**Affiliations:** Department of Social, Environmental and Economic Psychology, University of Koblenz and Landau, Landau, Germany

**Keywords:** coming out in long-term heterosexual relationships, parent–child communication, mixed-orientation marriage, straight spouses, disclosure

## Abstract

Which different feelings and reactions do different family members show if an adult family member who has long been perceived as heterosexual discloses their sexual identity as lesbian, gay, or bisexual (LGB)? Previous studies have investigated reactions of spouses and sometimes children in the United States. This article describes the findings of qualitative interviews and a German-language quantitative survey (*N* = 188) in which family members were asked about their emotions, experiences during the coming out process, and their use of support options. The samples were recruited via different LGB+ online forums and organizations in Germany, Austria, and Switzerland (age *M* = 44.08). The results demonstrate that a coming out after years of a heterosexual biography and family life affects adults’ parents and siblings in addition to spouses and children. Siblings were perceived as a supportive group of family members showing calm and mostly positive reactions. Parents mainly reported surprise but also showed on the one hand interest in supporting their children in the coming out process; on the other hand, we find also evidence for negative reactions including rejecting behavior. Children’s coping and well-being depended on the time that had passed since the coming out and their age at the time of the coming out. Spouses felt shocked, angry, and the ground was pulled from under their feet. Comparing the perspectives of these groups of family members, differences between them, and their specific needs are discussed. Practical implications are derived from the support options mentioned, and range from information from books, the internet to professional advice. Spouses report the need of diverse support options, whereas other family members ask friends and other family members for support. Forums, counseling and the possibility to exchange were perceived as a support but were more accessible for women than for men. They provide the chance for a communicative exchange before the stress becomes too strong.

## Introduction

A typical romantic love story is often depicted as follows: A man and a woman fall in love with each other, get married, move in together in their house, give birth to one or more children, and the couple lives happily ever after. The family is surrounded by grandparents and all other family members aspiring similar values for their lives. What feelings and reactions can follow, if after years one of the spouses discloses that he or she is gay, lesbian, or bisexual (henceforth, LGB+), or if family members find out about it? Some previous U.S.-based research has investigated straight spouses’ reactions. The present study widens the perspective to a different culture, German-speaking countries, and to other groups of family members: How do other family members, primarily children, parents, and siblings, feel and react, in addition to straight spouses?

The process of a coming out in a long-term heterosexual relationship can be very challenging not only for the involved gay men, lesbians, and bisexual women and men, but also for their family members because they are affected by discontinuities in established personal relationships, expectations of having a heterosexual relationship, and strong gender and family scripts (Buxton, 2000, 2012; Wolkomir, 2009; Hernandez et al., 2011; Li and Samp, 2019). In this study coming out in a long-term heterosexual relationship is defined as an identity development process later in life including the disclosure of gay, lesbian, or bisexual feelings to family members or partners after years of a heterosexual relationship (or marriage) with or without children (Hernandez et al., 2011). In other words, over many years the family system evolved, existed, and functioned in heteronormative ways, and suddenly a fundamental part of the family’s existence is put into question. Thus, coming out in a long-term heterosexual relationship is not only challenging because family members need to deal with their own thoughts and feelings and possibly negative attitudes toward homosexuality and bisexuality, but also because their established family and gender scripts and former family structure need to be re-adjusted toward an individual “solution” beyond established family scripts. This situation can be a crisis for the whole family system, and “neglecting one member of a family (…) during a family crisis, of course, impedes everyone’s healing and recovery” (Buxton, 2006, p. 318).

In spite of this observation, much less research has been done on the reactions and ways of coping of other family members but spouses. How did parents react when their son or daughter told them that they are in love with a new partner of the same gender? Which impact does a person’s coming out in a long-term heterosexual relationship have on their siblings? How did children handle that their mother or father decided to live as lesbian, gay, or bisexual in spite of the long-term relationship to their other parent?

To the best of our knowledge, different family members have never been examined in the same study. Only this kind of comparison would enable the analysis of differentiated reactions, coping strategies, and the adaptation of an adequate counseling process. This is what the present article aims at doing. With a quantitative analysis of the individual reactions of parents, siblings, children, and straight partners the study provides differentiated results about reactions and needs of these family members.

### Theoretical Models of Coming Out: Strategies and Reactions

When people implicitly assume that the person facing them is heterosexual, they take a heteronormative perspective (Herek, 1990). People assume heterosexuality unless the person communicates that he or she is lesbian, gay, or bisexual, which is summarized under the term *compulsory heterosexuality* (Manning, 2016). In many societies, negative attitudes and discriminatory behavior toward non-heterosexual people has existed and still exists today. At the same time lesbians, gay men, and bisexual people often find themselves in a situation in which they have to come out to others and disclose that they have romantic and sexual feelings toward members of the own gender (Rust, 2003). Different processes can lead to conversations in which one’s own sexual orientation is revealed. This can happen early in life, but also after people have already entered into a long-term heterosexual partnership. There is evidence that family members experience minority stress when a close relative reveals an LGB+ identity. This was very dominant for spouses (Reid et al., 2021). Negative feelings in the coping process are experiences of fear, stigma, prejudice, and discrimination. The minority stress model predicts that the expectation of being rejected, concealing one’s own sexual orientation, and internalized homonegativity can cause serious problems (Meyer, 2003). Family members reported that they concealed the coming out of their family members which implies the expectation of being rejected and internalized homonegativity. Positive effects of a coming out can be a positive growth, including a higher well-being and authenticity. Those individuals can develop a more positive LGB+ identity (Vaughan and Waehler, 2009).

A large body of research has focused on processes related to decisions to come out (for early models, see Cass, 1979; D’Augelli, 1994). A good background for the present study is the *constitutive coming out model* that provides a current understanding of how different levels (culture, cognitive processes, and interactions) and communication strategies are related to each other in coming out processes (Manning, 2014, 2016). It proposes coming out processes on three levels and predicts that any level can have a direct effect or impact on the other levels. First, on the cultural level people are informed about norms and viewpoints related to the acceptance of sexual orientation. Second, culture and people’s personal viewpoints affect whether and how they share their sexual identity with others. From the perspective of coming out in a long-term heterosexual relationship, a person may be perceived as being adapted to norms provided by culture and the family system. The coming out may violate these norms which may create a conflict for the acceptance of one’s own sexual orientation.

Third, on the individual level, one’s sexual identity as lesbian or gay could be formed within a process including several stages (Cass, 1979). The process can lead from the stage of confusion, when people realize that they do not have a heterosexual identity, through tolerance and acceptance, until they finally feel pride about themselves and their sexual orientation is synthetized into their identity (Cass, 1979). Even if other researchers (Sophie, 1986) pointed out that people do not need to go through all of the stages and that they may go back and forth, the model provides valuable explanations why people hesitate to disclose their LGB+ identity. Especially when coming out at a later age and a life plan has been established to be heterosexual, including marriage and children, people may not allow themselves to feel their homosexuality accepted and appreciated. Different predictors may promote or inhibit the decision to come out. The *model of sexual orientation disclosure decisions* predicts that there are interrelated factors (sexual identity development process, relationship dynamics, messages, expectations of outcomes) which influence the decision of non-heterosexual youth to disclose or not to disclose their sexual orientation to their parents (Grafsky, 2018). Similar processes can be expected if someone has a coming out in a long-term heterosexual relationship.

Equally important to processes that lead to conversations to reveal one’s sexual orientation are the consequences that such disclosure produces in family members. Generally, lesbian, gay, and bisexual individuals can experience stress when they fear experiencing, or directly experience, stigma, prejudice, and discrimination because of their sexual orientation. Coming out can have negative consequences such as physical threats, being fired from work, rejected from religious activities, and banished from families (Steffens and Wagner, 2009; Tilcsik, 2011; Katz-Wise and Hyde, 2012). For example, sexual minorities have a higher risk of cardiovascular diseases (Sherman et al., 2022). Because of these risks, people may decide to stay in the closet and hide their sexual orientation. The finding that minority stress has negative effects on depressive symptoms and self-esteem was replicated in an intersectional approach for sexual and gender minority adolescent of color (Mereish et al., 2022). Of high importance for one’s own mental health is social support and coping by family members as close relatives. They are able to buffer negative effects and support lesbian, gay, and bisexual individuals in achieving an integrative self-concept with their sexual identity. This support is particularly important both in adolescence and in adulthood, when a heterosexual relationship already exists and is followed by coming out.

### Reactions of Family Members Following Coming Out During Adolescence

Most research about the coming out process within families has focused on coming out during adolescence, taking the perspective of the gay, lesbian, or bisexual adolescent. If other family members were included in the analyses, these were mostly the parents. In most studies, young gay men or lesbians were asked about their parents’ reactions to their coming out (e.g., Cramer and Roach, 1988; Savin-Williams, 1989; Maguen et al., 2002; Steffens et al., 2010). However, some studies directly asked parents (e.g., Ben-Ari, 1995) or siblings (Jenkins, 2008; Osborn and Lugo, 2011) about their reactions. The reaction of parents often turned out to be very negative, including grieving the “loss of the child,” rejection, and feelings of guilt and failure (e.g., Strommen, 1989). However, other studies emphasized that the reaction of parents was particularly negative in the beginning and became more positive and supportive over time (e.g., Cramer and Roach, 1988). The child’s disclosure of a LGB+ identity affects parents’ identity and is a complex process which introduces new roles into the family system (Grafsky, 2014). Parents’ appraisal can be within a spectrum as lacking, negative, mixed, positive with silence, ambivalent, and validating. Open communication and harmony in the relationship are perceived as desirable (van Bergen et al., 2021).

Within the family, the relationship with siblings is also very important for adolescents. It is crucial for identity development, learning from each other, and exchanging about feelings and problems (Jenkins, 2008). The relationship between siblings can maintain its functions throughout the entire life. In the context of coming out, siblings are often the first person of trust. They may serve as reference of how the rest of the family will react and as a source of support (Jenkins, 2008; Osborn and Lugo, 2011). The reactions of siblings described in previous studies vary – as for parents – from very negative to positive and supportive. However, with siblings, studies emphasize more the supportive character of the relationship. In some cases, siblings can replace missing parental support.

As a coming out affects different family members, it affects the family as a whole and the analysis of the family’s adjustment is an important topic for research. In the context of coming out during adolescence several models of family adjustment have been developed which included the reactions, narratives, and coping strategies of different family members (e.g., Strommen, 1989; Beeler and DiProva, 1999; Crosbie-Burnett et al., 2006; Baptist and Allen, 2008). Previous models on family adjustments to coming out assume that families have to deal with different themes that do not follow specific stages of processing like former stage models stated (see Beeler and DiProva, 1999; Baptist and Allen, 2008). A study on discussions about homosexuality included themes such as seeking information about homosexuality and the LGB+ community, dealing with heteronormative conventions; working through first feelings of sadness, loss, and blame; coming out as a family; and stigma management (Beeler and DiProva, 1999). The authors emphasized that these themes occurred in an event-oriented way and that these challenges may be repeatedly revisited. Newer studies present evidence of positive effects of an improving social climate, social attitudes, and policies on well-being, especially for emerging adults (Frost et al., 2022).

### Reactions of the Family of Origin Following Coming Out Later in Life

Until today, models of family adjustment to coming out and research about the reaction of family members (especially parents and siblings) are confined to coming-out situations during adolescence. For coming out in long-term heterosexual relationships, it can be expected that the reactions of parents and siblings are as a rule comparable to those following a coming out during adolescence. However, in the situation of a coming out after years of a heterosexual family life there are other important family members that are immediately affected by the coming out: partners or spouses (henceforth referred to as spouses) and in many cases children. Furthermore, with a coming out in long-term heterosexual relationships there are new themes to deal with for the whole family system and individual family members (e.g., reorganization of the family structure following a potential separation of the mixed-orientation couple, concerns about the children, questions about the authenticity of relations). Hernandez et al. (2011) stated for the context of coming out in a long-term heterosexual relationship that the “perspectives of parents, in-laws, spouse, siblings, and children are largely missing in the literature” (Hernandez et al., 2011, p. 316). It is important to shed more light on them, before researchers and practitioners can develop family adjustment models for coming out situations in long-term heterosexual relationships involving all family members.

To our knowledge, no study has been carried out explicitly on the reactions of parents and siblings following a coming out in a long-term heterosexual relationship. The theoretical relevance of the present study can be derived from the personal importance of parents and siblings for individual identity because they are the closest members of one’s own family of origin. For example, entering into marriage and having children can fulfill (hetero-)normative expectations that parents have had for their children. Siblings are relatives with whom one shares one generation and they may have already fulfilled parents’ expectations within the family. Possibly, these expectations are broken with the coming out (Grafsky, 2014). Research projects that have used different methodological approaches show that siblings can be a resource in the coming out process: Different predictors as the relationship in adulthood, the contact to lesbian and gay individuals, the support of lesbian and gay rights, and religious attendance were related to siblings’ acceptance of lesbian and gay sisters and brothers (Hilton and Szymanski, 2014). Family pressure to marry and being bisexual were positively correlated with the internalized homonegativity in a sample in Lebanon (Maatouk and Jaspal, 2022). A qualitative comparative field study found that siblings offered socio-emotional support for their lesbian or gay sisters and brothers coming out. They were perceived as a confidential person when disclosing to their parents (Haxhe et al., 2018). A study, including an Italian sample, found that first-born gay men revealed their sexual orientation more likely to their fathers than gay men who had an older sibling; lesbians rather concealed their sexual orientation to their fathers and brothers (Pistella et al., 2020). Further, the internalized homonegativity moderated the effects of coming out to siblings. A low internalized sexual stigma was associated with a more likely coming out of gay men to their siblings (Salvati et al., 2018).

### Reactions of Spouses and Children Following Coming Out Later in Life

Most of the research body that focused on coming out in long-term heterosexual relationships has dealt with mixed-orientation-marriages (MOM) – marriages that consist of one straight person and one person with gay, lesbian, or bisexual feelings. Research about MOM has examined which predictors affect how the coming out message is communicated within the relationship (Li and Samp, 2020). The theory of coming out message production predicts that coming out processes in relationships are affected by different types of goals, which can be self-oriented, partner-oriented, or relationship-oriented (Li and Samp, 2019). For example, if individuals have the desire to maintain their heterosexual relationship, they may less likely come out to their partner or they disclose less information to minimize effect on their functioning relationship. But also, reactions, challenges, and themes of either one or both spouses (e.g., Hays and Samuels, 1989; Duffey, 2006; Li and Samp, 2021) following a coming out later in life, supporting and obstructive factors for MOM that decided to stay together (Buxton, 2000, 2004; Schwartz, 2012), as well as strategies that are pursued by these couples (e.g., Buxton, 2000, 2004; Wolkomir, 2009) have been in the focus of research. In general, there are different positive communicative behaviors in coming out conversations, such as direct affirmation, laughter, and joking, just as negative behaviors such as nervous non-verbal behavior, indirectly approaching the topic, expressing denial, religious talk, inappropriate questions, and shaming and aggressive statements (Manning, 2015). Recent studies provide evidence of minority stress in mixed orientation marriages for individuals coming out and their spouses (Reid et al., 2021).

Coming out in a long-term heterosexual relationship has also consequences on the decision whether the heterosexual couple separates or maintains their relationship. It has been estimated that around one third of MOM separate in the first year following the coming out (Buxton, 2006). Another third separates within the following 2 or 3 years while elaborating on different ways to maintain their relationship. The last third decides to continue the partnership or marriage – mostly with alternative concepts of relationships (e.g., open or polyamorous concepts). According to Buxton, around half of them stay together for three or more years.

Because most of the couples separate after the coming out, some of the themes the spouses deal with are similar to those for spouses after separation from a straight spouse (e.g., financial issues, visitations rights for children). In addition, there are several themes that are unique for straight spouses following the coming out of their spouses: uncertainty whether the relationship was authentic, feelings of guilt, questioning gender roles, questioning own sexual identity or orientation, concerns about the stigmatization of the family or the children, keeping homosexuality or bisexuality a secret, and fear of sexually transmitted diseases (see Buxton, 2006). For straight husbands a coming out of their wives can be related to a threat of their masculinity (Buxton, 2012; see Vandello and Bosson, 2013). The first emotional reactions of female spouses following the coming out of their husbands ranged from anger, grief, disgust, repulsion, or jealousy to feelings of relief in getting validation of their suspicions or relief about not being “guilty” for relationship problems (Hays and Samuels, 1989). As obstructive factors for the maintenance of the MOM, straight women cited their traditional concepts of marriage, their friends’ negativity, and their husband’s need for gay sex or a gay relationship (Buxton, 2000). In contrast, they cited the quality of the relationship and the well-being of their children as supportive circumstances for maintaining the MOM (Buxton, 2000). Further, mothers tend to come out later in a heterosexual relationship than non-mothers, because they take longer to identify as lesbian or bisexual (Morris et al., 2002). Far more rarely than straight wives, straight husbands have been the subject of research concerning their reactions toward the coming out of their wives. In one study Buxton (2004) examined the strategies that straight husbands and their lesbian or bisexual wives used in order to maintain their marriage. The most cited strategies by straight husbands were communication with their wife, counseling or therapy, and peer support (Buxton, 2004). As negative factors interfering with maintaining the MOM straight husbands cited perceived negative attitudes by their family of origin and perceived negativity by the lesbian and straight communities.

Children of these MOM are the next close relatives who are directly affected by a coming out. However, they have hardly been studied. In a German study, parents living in a same-sex relationship were surveyed about the reaction of their children when they learned about the homosexuality or bisexuality of the parent (Rupp, 2009). Results revealed that 23% of the parents reported their children were interested and 20% stated that their children liked it. “Interested” in that context implies that children ask their parents questions about their sexual orientation and how it may affect their life together. In this context, children can ask whether the parent has always been gay or lesbian, whether the parent is already in a new relationship, and why the coming out has occurred now.

Only 10% of the parents reported that their children were concerned about the reaction of their peers, whereas 27% of the children reacted ‘‘very emotionally.’’^1^ A study in South Africa, including eight interviews with adults who experienced their fathers’ coming out, could show that the coming out of a father can be interpreted as a disruption of a father figure that is considered to be a requirement in heteronormative societies. This can imply a negative stigma that leads to a higher awareness in gay fathers in terms of their public perception (Macleod, 2017). Overall, the gay, lesbian, or bisexual parent did not perceive their coming-out as having a more negative influence on their children than the separation of the parents did (Rupp, 2009). Taken together, reactions of straight husbands, children, and other family members to the coming out have rarely been studied and there is no single study which includes these different perspectives. This was the main purpose of our research.

### The Present Study

The present study aimed to give insights into the reactions and coping strategies of different groups of family members after a coming out in a long-term heterosexual relationship. The processing of the whole family system depends on the reactions of all family members – members of the nuclear family (i.e., spouses, children) and of the extended family (i.e., parents, siblings). As U.S.-studies showed reactions and coping strategies of spouses (mostly wives), the present study focused in particular on family members. Children, husbands, parents, and siblings were not in the focus of previous research which is our research goal. We test whether other groups of relatives than spouses and children show negative reactions after coming out. Wives were included for reasons of comparison. The interest of this study was in feelings, reactions concerning the coming out, and the use of support options in order to inform counselors and other practitioners about family members’ specific needs. Another purpose was to widen the methodological approaches that have been used to examine reactions toward coming out in long-term heterosexual relationships. Previous research mainly employed qualitative methods and assessed individual emotions, experiences, and narratives (e.g., analyzing interviews, self-reports, or counseling meetings) (e.g., Fruhauf et al., 2009; Grafsky, 2014; Nedela et al., 2021). The present study used a mixed-methods design with a qualitative part (eight interviews) and a subsequent quantitative part in which all individuals were asked to respond to the statements sampled during the qualitative part.

The present study covers German-speaking countries including Germany, Austria, and Switzerland, where “marriage for all” was introduced by law within the last few years (Germany: 2017, Austria: 2019, Switzerland: 2021). The study’s main innovative aspect is to obtain the reaction of different groups of family member in German speaking countries. Germany, Switzerland, and Austria represent western countries in which the social and political situation toward gender and sexual orientation minorities is in a transformation process between legal discrimination and acceptance. This means that these countries, after Scandinavia, France, and Spain, cannot be considered as pioneers in terms of equality policies for lesbians and gay men. In public life, politicians have been open about their sexual orientation, such as, in Germany, a gay mayor of Berlin (Klaus Wowereit), a gay Minister of Foreign Affairs (Guido Westerwelle), and a gay Minister of Health (Jens Spahn). Even if average religiosity is rather low, people of faith tend to show more negative attitudes toward lesbians and gay men than non-denominational people (Steffens and Niedlich, 2021).

## Materials and Methods

### Samples

In order to construct the questionnaire for the quantitative part of the study, telephone interviews were conducted with eight participants who brought in different perspectives. We interviewed one son of a woman who had come out, one father-in-law of a man, two female and two male straight spouses, as well as one woman and one man who had a coming out after a long-term heterosexual relationship and told us about the reactions of their different family members. All interview partners reacted to our announcement in online newsletters and online forums.

The subsequent questionnaire was filled out by 213 participants responding to our announcements in different LGB+ online forums, in the newsletter of the Lesbian and Gay Association Germany, in different online peer groups for female and male straight spouses, in counseling centers, and in the media (e.g., radio, newspaper). All participants who did not consent with the use of their data and all participants who did not fit the sample (e.g., individuals coming out in a long-term heterosexual relationship themselves) were excluded from analyses. Furthermore, in order to get partial samples, we excluded several individual participants who were neither children nor parents, siblings, or spouses of the people coming out (e.g., grandmother, cousin). Apart from that, there were no criteria for participation in order to promote an open and trusting atmosphere regarding this potentially sensitive topic. Participation was open to any person of legal age who felt invited via the link. The data collection was online. The duration of the survey was not recorded, but was estimated to be between 20 and 40 min. Participants received no compensation for their participation. All in all, the data of 188 participants (136 women, 52 men) were analyzed. Sample sizes of the partial samples are displayed in Table 1.

**TABLE 1:** Samples of participant groups in questionnaire study.

Sample:	Female (*n* = 136)	Male (*n* = 52)	Mean age of siblings, parents, children, and spouses (with *SD*)
Siblings (*n* = 49)	34 Sisters	15 Brothers	38.14 (12.30)
Parents (*n* = 32)	24 Mothers	8 Fathers	61.41 (14.30)
Children (*n* = 56)	37 Daughters	19 Sons	27.64 (9.14)
Spouses (*n* = 51)	41 Spouses	10 Spouses	49.14 (9.28)

Among the family members coming out, 77% came out as lesbian or gay (70% of women as lesbian, 83% of men as gay) and 18% as bisexual (no indication of sexual orientation was given by 5%). Among all participants, 175 were Germans, five came from Austria, and six from Switzerland (two did not indicate their nationality); 83 indicated to be Protestants, 48 Catholics, and 54 were atheists (three indicated “other religion”). Participants were predominantly well educated. The low participation rate of men was remarkable (28%; see Table 1). Particularly, the partial samples of fathers and male spouses were small. Therefore, the results of parents and spouses will not be presented separately for women and men. Previous research found that heterosexual men show more negative attitudes toward homosexuality than heterosexual women (Whitley, 2001; Herek, 2002; Tucker and Potocky-Tripodi, 2006; but for no robust effects, see Preuß et al., 2020). For children and siblings, possible gender differences were tentatively tested. In all partial samples, there was approximately the same number of female and male family members, except for the spouses: Whereas 41 female spouses reported about the coming out of their (ex-)husband, only 10 male spouses reported about the coming out of their (ex-)wive. The number of family members with coming out and number of years since the coming out are presented in Table 2.

**TABLE 2:** Number of family members with coming out and number of years since the coming out.

	Siblings	Parents	Children	Spouses
	
	% (*n*)	% (*n*)	% (*n*)	% (*n*)
Female family member	Sister: 49 (24)	Daughter: 47 (15)	Mother: 61 (34)	Wife: 20 (10)
Male family member	Brother: 51 (25)	Son: 53 (17)	Father: 39 (22)	Husband: 80 (41)

	***M* (*SD*)**	***M* (*SD*)**	***M* (*SD*)**	***M* (*SD*)**
	
Years since the coming-out	7.10 (6.22)	9.58 (7.86)	8.71 (5.73)	6.98 (7.08)

Among spouses, 20% indicated that they separated immediately after the coming out. From the remaining couples 59% separated 3 years (on average) after the coming out. The remaining ones – mainly straight female spouses with bisexual or gay husbands – still lived together as a couple and tried to work out appropriate concepts for their relationship (e.g., open relationships).

### Measures and Instruments for Data Gathering

The primary objective of the eight interviews was to identify possible strategies, challenges, thoughts, and themes people dealt with following the coming out situation, in addition to the knowledge we had gained from the cited literature. This was the first step to develop instruments for quantitative data gathering. Semi-structured interviews were conducted to gain an idea about feelings and reactions the family members experienced during the coming out process.

One prominent result in the interviews was that there are different reactions, themes to cope with, and challenges for spouses of people with a coming out as opposed to all other family members. In order to deal with these differences, two different versions of the questionnaire were constructed: one for spouses and one for all other family members. Some specific themes and variables to the spouses’ questionnaire were added which will not be discussed in the current article (e.g., gender identity-related variables) because this article focuses on the comparison of different groups of family members.

In the second step, the quantitative survey was constructed and included the following sections: After informed-consent information, the questionnaire assessed socio-demographic information (e.g., age, nationality, religion, education). Participant were asked about their sexual orientation on a scale to be able to capture the whole spectrum of different sexual orientations (1 “lesbian/gay” to 7 “heterosexual,” “bisexual” as 4 in between). For gender, the options “male” or “female” were used. We further provided the answer “other” gender identities using an open field.

We mainly used non-standardized questions that we developed on the basis of the qualitative interviews. If standardized measures were used, we cite the source in the text. The topics were: first emotional reactions (e.g., “I was relieved/surprised/sad.”; dichotomous scale with *yes/no* and multiple answers possible), thoughts, and strategies following the coming out (e.g., “My worldview was turned upside down.”, “I kept the coming out a secret.”, “I was very happy for her/him.”, 7-point Likert-scale from 1 *This is true* to 7 *This is not true*). Participants were asked about participants’ actual well-being (Dalbert, 1992; seven items on a 7-point Likert-scale from 1 *do not agree at all* to 7 *fully agree*; e.g., “Most of the time I am feeling very happy,” “When I look back on my life, I am very satisfied.”; Cronbach’s α for all family member groups from 0.84 to 0.95). It was further assessed the quality of relationship with the gay/lesbian/bisexual family member (one item with response options *worse/same/better*; “Did the relationship with the family member with coming out change?”) and thoughts and attitudes toward the coming out (e.g., “The coming-out had a big influence in my life.”, “I was not very touched by the coming out.”; 7-point Likert-scale from 1 *do not agree at all* to 7 *fully agree*). The questionnaire also included present attitudes toward lesbians and gay men (Simon, 2008; four items on a 7-point Likert-scale from 1 *not agreed at all* to 7 *fully agree*; e.g., “It is disgusting when two men kiss in the street.”, “If I were aware that my neighbor is a lesbian, I would avoid contact.”; α for all family member groups from 0.68 to 0.95). In the final section, we asked participants whether they made use of support options (e.g., online social networks, talking with friends, talking with family, self-help groups, individual life counseling, therapy; dichotomous items with *yes/no* and multiple answers possible). Due to different topics that were identified in the interviews for spouses versus others, participants were assigned to one of two questionnaire versions. A filter function directed participants to either the “partner questionnaire” or the “family member- questionnaire.” When thanking them for their participation, we collected consent to use their data, now that participants were well-informed about all detailed questions.

### Procedure

For the qualitative part of the current research, the questions were non-standardized, but we asked for a detailed answer. We asked participants to tell the story about the coming out, which feelings were present in the beginning and how they developed over time. The relationship to the family member having the coming out and how it developed over time were further topics. The choice of interview questions was guided by the research question of the research project. To participate in an interview, we used criteria that were similar to those used in the survey, according to which interviewees had to be at least 18 years old and a sibling, parent, child, or (former) partner of someone who had a coming out in a heterosexual relationship. Interviews were conducted by telephone after making an appointment.

Individuals who participated in the quantitative survey, gained access via an online link. Depending on whether they were the partner or a family member of a person who had a coming out in a heterosexual relationship, they were assigned to one of the two questionnaires. They were guided through the different questions of each topic. Participants had the option of not answering questions and all questions were presented in the same order. They had the possibility to stop the survey at any time. Furthermore, our contact address and the contact of a counseling center were displayed, in case of a need of consultation. Finally, they were thanked for their participation.

We followed all ethical guidelines and the study was reviewed and approved by Ethics Committee of the Friedrich-Schiller-Universität Jena. The participants provided written informed consent.

In general, the sample is characterized as a convenience sample, including a low proportion of men; especially husbands are underrepresented. For this reason, we can only interpret very substantial differences between groups of family members. Responses obtained in the interviews are interspersed among the quantitative findings below for illustration.

## Results

In the following, we summarize results on the broad topics. In order to illustrate the findings more clearly, they are presented with a quotation for each specific theme.

### Coming-Out Situation

Averaged over all participants, the coming out was 8 years ago (see Table 2). Thus, participants were willing to participate in the surveys years after the coming out, and only few individuals provided information about their feelings and reactions shortly after. In 81% of all cases, the family members learned about the homo- or bisexuality by those coming out themselves (e.g., in a personal conversation); 19% learned about it in a different way (e.g., deducing it from behavior).

### First Emotional Reaction and Thoughts

As first emotional reactions after they learned about the homo- or bisexuality of their family member participants reported a variety of different emotions. More than 30% of all partial samples (parents, siblings, spouses, and children) indicated that they were surprised about the coming out. In particular, children were surprised when their mother or father told them that they were gay, lesbian, or bisexual. The comparison of all patterns of emotions for the parents, siblings, spouses, and children clearly showed a qualitative difference between the emotional reactions (see Table 3).

**TABLE 3:** First emotional reaction immediately after the coming out.

	Siblings	Parents	Children	Spouses
				
I was…	% (*n*)	%(*n*)	% (*n*)	% (*n*)
Relieved	**29 (14)**	19 (6)	13 (7)	22 (11)
Angry	4 (2)	13 (4)	18 (10)	14 (7)
Furious	0	3 (1)	13 (7)	**37 (19)**
Sad	14 (7)	**31 (10)**	**25 (14)**	**59 (30)**
Lonely	2 (1)	3 (1)	16 (9)	**31 (16)**
Shocked	6 (3)	22 (7)	**27 (15)**	**67 (34)**
Desperate	2 (1)	19 (6)	13 (7)	**59 (30)**
Fearful	6 (3)	9 (3)	**25 (14)**	**31 (16)**
Helpless	14 (7)	**38 (12)**	**32 (18)**	**65 (33)**
Unconcerned	12 (6)	0	5 (3)	0
Surprised	**45 (22)**	**41 (13)**	**68 (38)**	**37 (19)**
Composed	20 (10)	19 (6)	**27 (15)**	14 (7)
Happy	**41 (20)**	13 (4)	13 (7)	0
Interested	**57 (28)**	**41 (13)**	**29 (16)**	12 (6)
Calm	**27 (13)**	13 (4)	11 (6)	2 (1)
Without emotions	4 (2)	3 (1)	9 (5)	10 (5)

*Bold printed values indicate that more than a quarter of the sample stated that feeling.*

Parents reported that they were mainly surprised, but also interested in the coming out of their adult children. Many indicated that they were helpless and sad in that situation. In contrast to all other partial samples, siblings reacted mainly in positive ways to the coming out. They reported next to surprise that they felt interested, happy, relieved, and calm. The fact that 22 siblings indicated to be gay, lesbian, or bisexual themselves may have played a role in their quite positive reactions. Taking only the 27 straight siblings into account revealed that they were also mostly surprised (52%) and interested (52%). Also, about one third of the straight siblings reacted happily and took the coming out with calm. However, another third of them also indicated to be sad and helpless.

Next to surprised, children felt rather helpless. However, they also reported that they were interested (29%). The reactions of the children seemed to be quite diverse. Whereas 27% reported that they were shocked by the news of the coming out, also 27% reported that they took it with calm. One quarter of the sample felt sad and fearful.

We ran chi-square analyses to assess differences family members’ first emotional reactions during the coming out (but see Footnote 1 to avoid over-interpretation). We ran these analyses for those feelings that at least one quarter of a group reported. Rather positive feelings of relief (*p* > 0.05), happiness [χ2(3, *N* = 188) = 32.15, *p* < 0.001, *V* < 0.001], and calmness [χ2(3, *N* = 188) = 13.90, *p* < 0.01, *V* < 0.01] were reported significantly more often by siblings than by parents, children, and spouses. Most of the siblings, parents, and children felt interest as a first emotional reaction immediately after the coming out, which was only reported by less than one quarter of spouses [χ2(3, *N* = 188) = 24.45, *p* < 0.001, *V* < 0.001].

Negative feelings of being sad [χ2(3, *N* = 188) = 24.99, *p* < 0.001, *V* < 0.001] and helpless [χ2(3, *N* = 188) = 28.13, *p* < 0.001, *V* < 0.001] were reported mostly by parents, children, and spouses, but less by siblings. Children and spouses as the closest relatives in the current relationship reported feelings of being shocked [χ2(3, *N* = 188) = 45.90, *p* < 0.001, *V* < 0.001] and being fearful [χ2(3, *N* = 188) = 13.51, *p* < 0.01, *V* < 0.01]; and only children reported the feeling of being composed [χ2(3, *N* = 188) = 45.90, *p* < 0.001, *V* < 0.001]. Only descriptively spouses but not siblings, parents, and children reported feelings of loneliness and desperateness [χ2(3, *N* = 188) = 2.88, *p* = 0.411, *V* = 0.411]. All groups of family members indicated being surprised, and we found the highest number in children [χ2(3, *N* = 188) = 12.01, *p* < 0.01, *V* < 0.01].

A narrative of a son whose mother had a coming out illustrates personal feeling of anger, sadness, and helplessness, but also suffering from his parents’ divorce: “*I was mad that my mother wants to leave. When she said that she lives with a woman, I was mad again. How long have you kept this from me now? The anger was more directed at the separation, and then came great insecurity, because I was suddenly afraid that I no longer knew my own mother*.” (son, 27 years old)

The emotional reaction of the spouses was the fiercest of all partial samples. Over 60% (46% of men) indicated that they were shocked and almost all felt helpless in the first place. Mostly, spouses were desperate and sad. In contrast to the other partial samples, spouses also reported that they felt lonely and very angry. Only a small minority indicated to be “interested” (12%; 15% of men). Spouses also suffered from questioning their own relationship and life plan, as one male interviewee illustrates: “*It really gnaws at your self-confidence, also at your sexual identity, to know that my partner is giving me up for a partner of the same gender. I thought things like: You failed as a husband. You are nothing. I also blamed myself for that*.” We summarize the first emotional reactions immediately after the coming out in Table 4.

**TABLE 4:** Affirmation of statements about thoughts right after the coming out.

	Siblings	Parents	Children	Spouses
	
	% (*n*)	% (*n*)	% (*n*)	% (*n*)
My world was turned upside down.	2 (1)	17 (5)	**25 (14)**	**51 (26)**
I kept the coming out a secret.	14 (7)	21 (6)	**38 (21)**	**45 (23)**
The situation was embarrassing for me.	4 (2)	23 (7)	17 (9)	**29 (15)**
I thought others would get a negative impression of me.	4 (2)	10 (3)	20 (11)	24 (12)
I was very happy for her/him.	**60 (29)**	**41 (12)**	**25 (14)**	6 (3)
I was sure I would support the person coming out.	**67 (33)**	**75 (24)**	**50 (28)**	29 (15)
I had the feeling, everything that I hoped for the life of that person was gone.	2 (1)	16 (5)	5 (3)	N.a.

*Affirmation for a statement was defined as the values 1 and 2 on a 1–7 scale. Values printed in bold indicate that more than a quarter of the sample affirmed the statement.*

*“N.a.”, not asked.*

In order to get a more complete picture of first emotional, cognitive, and behavioral reactions of family members, we asked participants to indicate retrospectively whether they affirm or disaffirm specific statements (for a selection of statements see Table 4). Only few siblings and parents felt that the coming out “turned their world upside down.” In contrast, the majority of the siblings (60%) and nearly half of the parents were happy for the person with the coming out. However, half of all spouses and one quarter of children perceived the situation as “turning their world upside down.” Nearly half of all spouses kept the homosexuality or bisexuality of their partners a secret, at least in the beginning, and only 6% stated they had been happy for the person with the coming out. As reasons for this coping strategy one straight wife mentioned in her interview the fear that an official coming out to others may lead to divorce and her wish to stick with the family. Some spouses also indicated in the questionnaire that they felt embarrassed. An interviewed spouse whose wife had a coming out articulates his shock: “*I was totally exhausted. I don’t even remember if I yelled or if I screamed. Then I went out and she followed me because she was afraid, I would hurt myself*. (…) *Later I went to the doctor and he admitted me to the psychiatric ward*.” In line with this pattern of results is the finding that 67% of the siblings and three quarters of the parents supported the family member with coming out in the heterosexual relationship without questioning, whereas only 29% of spouses affirmed this statement. Of all family members, only spouses but not siblings, parents, and children felt embarrassed right after the coming out [χ2(3, *N* = 188) = 11.60, *p* < 0.01, *V* < 0.01]. Siblings, parent, and children, but not spouses affirmed the statements that they could be happy for the person coming out [χ2(3, *N* = 188) = 34.90, *p* < 0.001, *V* < 0.001] or were sure they would support the person coming out [χ2(3, *N* = 188) = 21.87, *p* < 0.001, *V* < 0.001]. More than one quarter of children and spouses described their thoughts right after the coming out as if the world was turned upside down [χ2(3, *N* = 188) = 34.10, *p* < 0.001, *V* < 0.001] and they kept the coming out a secret [χ2(3, *N* = 188) = 14.63, *p* < 0.01, *V* < 0.01].

### Present Well-Being

At the time of the study, most participants felt that they had found an appropriate way of coping with the coming out. Overall, they stated good well-being and high satisfaction with their lives in general (see Table 5). For parents and siblings, there was no relationship between well-being and coping depending on how long ago the coming out was (tested in a linear regression model). We ran this linear regression for the sample of children and found that their state of coping with the situation depended on the time that had passed since the coming out [β = 0.25, *t*(51) = 1.97, *p* = 0.05] and their age at the time of the coming out [β = –0.32, *t*(51) = –2.52, *p* = 0.02; *F*(2,51) = 6.80, *p* = 0.002, *R*^2^ = 0.21]. The longer ago the coming out was and the younger the children were at the time, the better they evaluated their coping with the situation. The same factors influenced their general well-being. The longer ago the coming out was [β = 0.35, *t*(51) = 2.71, *p* = 0.01] and the younger the children were at the time of the coming out [β = –0.25, *t*(51) = –1.99, *p* = 0.05], the more satisfied they were with their lives [*F*(2,51) = 7.49, *p* = 0.001, *R*^2^ = 0.23].

**TABLE 5:** Present well-being (seven items, Dalbert, 1992) and state of coping with the situation.

	Siblings	Parents	Children	Spouses
**Present well-being** ***M* (*SD*)**	5.22 (1.03)	5.36 (1.30)	5.50 (0.91)	4.68 (1.41)
	**% (*n*)**	**% (*n*)**	**% (*n*)**	**% (*n*)**
	
Low (1–2)	0	6 (2)	0	8 (4)
Middle (3–5)	**64 (32)**	**40 (13)**	**49 (27)**	**64 (32)**
High (6–7)	**34 (17)**	**53 (17)**	**52 (29)**	**30 (15)**
Cronbach’s α	0.95	0.95	0.84	0.91
**Coping with the situation** ***M* (*SD*)**	6.49 (0.92)	6.00 (1.63)	6.36 (1.31)	5.39 (1.82)
	**% (*n*)**	**% (*n*)**	**% (*n*)**	**% (*n*)**
	
I just started. (1–2)	0	0	4 (2)	14 (7)
Neutral (3–5)	14 (7)	19 (6)	13 (7)	**26 (13)**
I found an appropriate way of coping. (6–7)	**84 (42)**	**78 (25)**	**85 (47)**	**62 (31)**

*Bold printed values indicate that more than a quarter of the sample affirmed the statement.*

Also, for the spouses the time since the coming out had an influence on their well-being and their coping with the situation. The longer ago the coming out was, the better they evaluated their coping [β = 0.57, *t*(48) = 4.68, *p* < 0.001]. Their age at the time point of the coming out had no effect [β = 0.10, *p* > 0.05; *F*(2,48) = 10.98, *p* = 0.001, *R*^2^ = 0.31]. They rated their well-being more positively, the longer ago the coming out was [β = 0.36, *t*(48) = 2.63, *p* = 0.01]. Age had no influence [β = –0.05, *p* > 0.05; *F*(2,48) = 3.84, *p* = 0.003, *R*^2^ = 0.14]. Table 5 illustrates the family members’ present well-being and the state of coping with the situation.

### Quality of Relationship

The results revealed that the coming out was said to improve the relationship between the participating family member and the family member with coming out in many cases (see Table 5). The questionnaire for spouses did not include this question. In particular, parents reported that they had a better relationship with their gay, lesbian, or bisexual adult children following the coming out. In spite of the generally positive reactions of siblings, the relationship to some siblings was described as difficult to this day by a 68-year-old man whose coming out was 18 years ago: “*My brother, today 72 years old, does not want to talk about the fact that I am gay even today. He’s embarrassed when I tell him that. My sister doesn’t like to talk about it either. This gay side of mine is still very difficult for her*.”

Only a quarter of all children reported such an improvement of their relationship, with siblings’ scores in between (see Table 6). The son of a parent who had a coming out in a heterosexual relationship highlighted the importance of mutual communication as a personal resource:

**TABLE 6:** Change of relationship quality following the coming out.

	Siblings	Parents	Children
**Change of the relationship** ***M* (*SD*)**	5.35 (1.37)	5.77 (1.45)	4.47 (1.72)
	**% (*n*)**	**% (*n*)**	**% (*n*)**
Worse (1–2)	2 (1)	0	13 (7)
Same (3–5)	**48 (24)**	**39 (10)**	**46 (26)**
Better (6–7)	**42 (21)**	**62 (16)**	**25 (14)**

*Bold printed values indicate that more than a quarter of the sample indicated the option.*

“*Most of all I would argue mutual communication, joint activities, and awareness are of importance. The relationship continues even though it has changed. It has changed a little, but what you have together since, that continues*.” In Table 6, we summarize how family members perceived the change of relationship quality following the coming out.

### Use of Support Options

Children and siblings did not use as many different support options as compared to parents and spouses. For them, particularly the exchange with friends, other family members, and the person with the coming out were often used as support (see Table 7). Noticeably, parents and spouses used more different support options. Spouses used the whole variety of available support options. Parents searched much information about homosexuality – often for the first time in their lives – in books, the internet, and on television. However, also talking to friends, to their adult child who had the coming out, and to other family members was important support for them. More than other subsamples they also used the exchange with peers, in that case other parents in the same situation. Spouses also searched for information in books, brochures, and the internet – in particular in online social networks. Mostly, spouses talked to close friends (75%) but also to their homo- or bisexual partner, other family members, and peers. Noticeably, spouses took advantage of professional support more than all other subsamples. In particular, they went to individual counseling and couple therapy. Overall, talking with friends, the person coming out, and family members were the support options that were used by all groups of family member. Parents and spouses made rather use of support options that provide knowledge as books/brochures [χ2(3, *N* = 188) = 31.60, *p* < 0.001, *V* < 0.001] and the internet. Spouses used online social networks [χ2(3, *N* = 188) = 23.41, *p* < 0.001, *V* < 0.001] and support groups more often than siblings, parents, and children [χ2(3, *N* = 188) = 24.99, *p* < 0.001, *V* < 0.001]. Only parents but not siblings, children, and spouses used television (e.g., documentaries) as a source of information [χ2(3, *N* = 188) = 10.61, *p* < 0.01, *V* < 0.01]. Only spouses, but not parents, siblings, or children made use of individual [χ2(3, *N* = 188) = 27.03, *p* < 0.001, *V* < 0.001] or couple counseling [χ2(3, *N* = 188) = 38.63, *p* < 0.001, *V* < 0.001]. In the interviews the personal importance of professional support was articulated by a spouse: “*I just didn’t feel understood enough in a couple counseling with my wife. Twelve years later, affected men still tell me today: I can’t find anyone who suits my needs and could advise and accompany me.*” The support options siblings, parents, children, and spouses made used of are presented in Table 7.

**TABLE 7:** Use of support options.

	Siblings	Parents	Children	Spouses
	
	% (*n*)	% (*n*)	% (*n*)	% (*n*)
Books/brochures	22 (11)	**34 (11)**	5 (3)	**53 (27)**
Knowledge transfer via internet	20 (10)	**31 (10)**	9 (5)	**49 (25)**
Online social networks	16 (8)	16 (5)	7 (4)	**51 (26)**
Talking with friends	**51 (25)**	**47 (15)**	**61 (34)**	**75 (38)**
Talking to the person coming out	**63 (31)**	**59 (19)**	**48 (27)**	**39 (20)**
Talking with other family members	**41 (20)**	**41 (13)**	**45 (25)**	**35 (18)**
Television (e.g., documentaries)	18 (9)	**25 (8)**	13 (7)	2 (1)
Support groups	2 (1)	6 (2)	2 (1)	**49 (25)**
Talking with peers	18 (9)	**31 (10)**	7 (4)	**29 (15)**
Individual counseling	0	6 (2)	5 (3)	**29 (15)**
Couple counseling	0	3 (1)	2 (1)	**31 (16)**
Psychotherapy	2 (1)	3 (1)	7 (4)	14 (7)
Psychiatric hospital	0	0	2 (1)	8 (4)

*Bold printed values indicate that more than a quarter of the sample indicated the support option.*

### Attitudes Toward the Coming Out and Toward Gay Men, Lesbians, and Bisexual Women and Men

Siblings and parents affirmed that they supported the person with coming out the best they could. Children indicated that the coming out had a substantial influence on their lives, more than siblings and parents did. We illustrate these findings in Table 8 and give an overview about thoughts about the coming out and its impact on the participant and family. However, more than a quarter of the children also indicated that they were not very touched by the coming out. Children – although a bit less than parents and siblings – also supported their parents following the coming out. Over 80% of the children, siblings, and parents expressed that they were tolerant concerning different life styles (“Everybody should be free to live the way he or she thinks it’s right.”). They also reported that their families had become more open toward homosexuality and bisexuality following the coming out. Overall, we compared the answers of parents, siblings and children for each statement that at least one quarter of one group of family members agreed to Table 8. We found no significant differences for parents, siblings, and children for the statements “The coming out had a big influence on my life.” (*p* > 0.05), “I supported the person with coming out the best way I can.” (*p* > 0.05), “Everybody should be free to live the way he or she thinks it’s right.” (*p* > 0.05), and “My family has become more open toward homosexuality and bisexuality.” (*p* > 0.05). In the interviews, also negative attitudes were expressed, as an interviewee described his father: “*So with my father I think, even if he would never say so, he has a very clear aversion to gay men, because he cannot understand that, but also because he […] has never dealt with the issue*.” Nearly all study participants reported positive attitudes toward lesbians and gay men (see a summary of attitudes toward lesbians and gay men in Table 9).

**TABLE 8:** Thoughts about the coming out and its impact on the participant and family.

	Siblings	Parents	Children	Spouses
	
	% (*n*)	% (*n*)	% (*n*)	% (*n*)
The coming out had a big influence on my life.	**25 (12)**	**27 (8)**	**41 (23)**	Not asked
My life changed for the worse after the coming out.	0	3 (1)	7 (4)	Not asked
I was not very touched by the coming out.	13 (6)	14 (4)	**27 (15)**	Not asked
My image of the person coming out changed completely.	6 (3)	7 (2)	9 (5)	Not asked
I supported the person with coming out the best I can.	**71 (34)**	**77 (23)**	**59 (31)**	Not asked
Everybody should be free to live the way he or she thinks it’s right.	**86 (42)**	**84 (25)**	**88 (49)**	Not asked
It would have been better, if the person had not come out.	2 (1)	7 (2)	4 (2)	Not asked
The coming out was a “black chapter” of our family life.	2 (1)	10 (3)	6 (3)	**34 (17)**
My family has become more open towards homosexuality and bisexuality.	**40 (19)**	**53 (16)**	**34 (18)**	20 (10)

*Bold printed values indicate that more than a quarter of the sample affirmed the statement.*

*^2^These questions have not been asked to spouses for the reason that these issues can probably be too sensitive and too emotional for spouses. Basically, it can be perceived as provocative to ask whether a spouse feels “touched” by the coming out of the own partner and to which degree it changed the own life-plan. The focus on the research question was kept on how parents, siblings, and children react to these issues. For Spouses, we limited the questionnaire to emotions and the other variables we addressed.*

**TABLE 9:** Attitudes toward lesbians and gay men.

	Siblings	Parents	Children	Spouses
**Attitudes toward Lesbian and Gay** ***M* (*SD*)**	6.24 (1.41)	5.88 (1.39)	6.24 (1.10)	5.94 (1.12)
	
	**% (*n*)**	**% (*n*)**	**% (*n*)**	**% (*n*)**
	
Negative (1–2)	0	0	0	2 (1)
Neutral (3–5)	**29 (14)**	23 (7)	21 (12)	**32 (16)**
Positive (6–7)	**71 (35)**	**76 (23)**	**79 (44)**	**68 (34)**
Cronbach’s α	0.62	0.64	0.66	0.44

*Bold printed values indicate that more than a quarter of the sample held these attitudes.*

## Discussion

The purpose of the present study was to obtain an overview of the reactions and ways of dealing with coming out in a long-term heterosexual relationship from the perspectives of siblings, parents, children, and spouses. Family members assume *compulsory heterosexuality* until the person in the heterosexual relationship comes out as lesbian, gay, or bisexual (Manning, 2016). The findings show clear differences between the reactions of these groups of family members. Every family member thus has different themes to work on and uses different coping strategies to handle the situation. In comparison with the other groups of family members, siblings are rather characterized by positive feelings (relieved, happy, interested), while spouses show more negative feelings (furious, sad, fearful, helpless). Children report feelings of sadness, shock, fear, and helplessness, which are more similar to the feelings of spouses than to feelings of siblings. Parents report a variety of positive and negative feelings, such as sadness, helplessness, and surprise, but also interest.

It can be concluded that similar to findings regarding youth coming out (Jenkins, 2008; Osborn and Lugo, 2011), the most positive reactions were found for siblings who reacted mostly positive to the coming out in the long-term heterosexual relationship. This supports the findings of recent studies which provided evidence that siblings provide an emotional and social support and an accompaniment in the coming out process toward parents (Haxhe et al., 2018). We find less evidence of negative reactions of siblings, as recent studies had shown for older siblings and brothers (Salvati et al., 2018; Pistella et al., 2020). They often reacted calmly and expressed feelings such as surprise, interest, happiness, and relief. The sample included a higher number of siblings who were lesbian, gay, or bisexual themselves. This can be an asset for individuals who come-out in a long-term heterosexual relationship, because their brothers or sisters have already experienced the coming out process and can be a supportive source concerning upcoming questions and problems. Counseling centers may motivate lesbian, gay, and bisexual individuals who aim to have a coming out to inaugurate their siblings first and to talk about difficult reactions that may appear within the family.

Parents mainly reported surprise, but also interest in supporting their adult children and the family to get through the coming out process. There was evidence for very negative reactions, including grieving, the “loss of a child,” rejection, and feelings of guilt and failure, in line with previous research on family members’ reactions to youth coming out (e.g., Strommen, 1989). Parents’ reactions can thus be summarized as being in the spectrum of positive and negative reactions (van Bergen et al., 2021). We find evidence that helplessness and sadness about the situation were caused by a lack of experience with non-heteronormative family structures and internalized expectations of a normative biography. As the present and other studies with youth coming out found for most family member groups, the relationship became negative after the coming out but turned to be positive over time (e.g., Cramer and Roach, 1988). In these families also the parents go through different stages of identity formation as being the mother of a lesbian, gay, or bisexual child. As the findings demonstrate, the adult age of a child does not preclude parents’ need to adapt to the coming out. In general, parents, more than other family members make use of collecting knowledge and personal exchange to adapt to the new non-heteronormative situation. Here we should not disregard that not all adult children disclosed their sexual orientation to their parents, because they expected very negative reactions. This might happen in families in which the pressure to disclose one’s own sexual identity is low, in line with the predictions made in the model of sexual identity development process (Grafsky, 2018). Further, in the present study there was evidence of a personal growth not only for the person coming out but also the well-being and authenticity of family members who can develop a positive identity (Vaughan and Waehler, 2009). However, note that we were not able to include groups with very negative attitudes in the survey because they would decline participation.

As first emotional reaction, mostly children reported that they were surprised. Whereas many felt helpless, shocked, and fearful, others indicated that they were interested in that situation and would support the family member coming out. The younger the children were and the longer ago the coming out was, the better they dealt with the situation, and they reported high well-being in their lives at the time of the study. These findings are comparable to those previously reported (Rupp, 2009). Over one third of all children kept the coming out a secret at first. Thus, there is evidence in the data that different factors are of relevance also for children. As noted in the model of sexual identity development process (Grafsky, 2018), the nature of relationships (e.g., with friends, school mates), the expectations, and whether they feel high or low pressure to talk about their parents’ sexual orientation influence their strategies of dealing with the situation.

As support in that situation, most children preferred to talk to the person coming out, to other family members, and to friends. A very important issue for children – and in some cases the more important issue – was dealing with the potential separation of their parents. One important finding of the study was that children suffer more from the parental separation and family breakup than from the disclosure of their mother’s or father’s sexual orientation. Children also suffer from the fear that they will be the target of bullying because of parent’s coming out, which is in line with previous studies (Rupp, 2009). However, solutions for this problem could be provided through broad education about homosexuality and non-heteronormative perspectives. On a positive note, it can be emphasized that children who have experienced a parent’s coming out often report an intensifying parent–child relationship and positive attitudes toward homosexuality.

(Female) spouses were included in the study for reasons of comparison. For spouses the emotional reactions were described as a shock, anger, helplessness, desperation, and sadness, which can be illustrated as the feeling that the ground was pulled out from under their feet. Spouses may experience the highest breach of expectations of compulsory heterosexuality toward their partner because they share a romantic and sexual relationship with them. The interviews indicated that reactions of spouses were accompanied by questioning the relationship, their sexuality, and thereby their own life plan and identity. Others reported a feeling of relief after the coming out of their spouses because they felt something had changed their relationship. As a consequence of the coming out, many couples keep the sexual orientation of the gay, lesbian, or bisexual partner a secret for a while. Men whose wives had come out as lesbian or bisexual showed little willingness to talk about their experiences; at the same time, there are hardly any forums and opportunities for exchange with other men with comparable experiences. For the counseling and therapy of spouses, we can conclude that they are in the most difficult situation, compared with other groups of family members. Spouses can be in a situation in which they have no chance to win back their partners. They need support in redefining their life plan and to adapt in the new situation.

As it was the case in the relationship formation with the children, the relationship can improve again, the more time passes. There were also couples who decided to continue their marriage. Some couples maintain their marriage and agree to alternative forms in which same-sex relationship are integrated (e.g., open relationship). All in all, we replicate the finding that separations are similar to separations from a heterosexual partner (Rupp, 2009). As in previous research the present study provides evidence that a wife’s coming out was perceived as a threat to their masculinity for straight husbands (see Buxton, 2006). The hard time we had recruiting heterosexual men to participate in the study could be an indicator of such masculinity threat (Vandello and Bosson, 2013). For female spouses the variation between anger, grief, disgust, and relief that Hays and Samuels (1989) reported.

From a broader perspective, it can be concluded that a coming out in a long-term heterosexual relationship affects members of the whole family system. Positive effects of the social climate, social attitudes, and policies on well-being, especially for emerging adults and their family system can result in positive effects (Frost et al., 2022). It was not possible not identify specific stages of family adjustment as previous models postulated (see Beeler and DiProva, 1999; Baptist and Allen, 2008). There is evidence that family members feel confusion when a close person comes out in a long-term heterosexual relationship. Working through feelings such as sadness, loss, and blame during the coming out as a family is an essential part of stigma management (Beeler and DiProva, 1999). Common processes for the families are that they seek information and contact to lesbian, gay, or bisexual communities. This is a source to reflect own heteronormative conventions and attitudes.

### Limitations and Future Research

Several limitations of the present study need to be discussed. The individual processing and reactions of people of the coming out situation in the family can be very different. Whereas some people exchange their thoughts and feelings from the first day they learned about the coming out, others push the issue out of sight and would never take part in a study about coming out in a heterosexual relationship. In the study, all participants decided voluntarily to participate. Thus, it is in the nature of the study that the results of a representative sample that reflects all types of reactions of family members experiencing a coming out in their family cannot be reported. There was evidence for extremely negative forms of adjustment (e.g., breaking contact, negating). Because of the convenience sample, only very substantial differences between groups of family members can be interpreted. For this reason, we cannot make statements about people who have decided against disclosure because social norms do not allow them to come out or because they fear negative reactions, such as physical threat, being fired from work, or rejection for religious reasons (Steffens and Wagner, 2009; Manning, 2016). Because this group stays “in silence,” it cannot be assessed which norms prevail in the family system and how they can be positively changed.

In future research on coming out in long-term heterosexual relationships, it is important to focus not only on the perceptions and reactions of family members, but also on the individuals coming out. What are the causes for today’s coming out in a heterosexual relationship? Does the length of the heterosexual relationship affect when coming out occurs and how it proceeds? How do people with a coming out in a long-term heterosexual relationship experience the hide-game which can have lasted for years, the process of redefinition, but also the coming out into a new community?

Future research could focus on a broader perspective on reactions and processes in families with a coming out in a long-term heterosexual relationship. For example, less is known about coming out processes of older LGB+ people. First, there is evidence that older lesbians and gay men are perceived as invisible groups, which may be accompanied with suffering from isolation and loneliness in older age (for a similar line of argument, see Altman, 1999). Second, numerous older men were forced to a conversion “therapy” in their younger ages. The repression of their own homosexuality and internalized homonegativity can have serious consequences on the mental health and the well-being of individuals (for empirical evidence, see Lehavot and Simoni, 2011; Frost et al., 2015). A research questions of high relevance is to examine which factors motivate people to decide for an open and positive approach with their own homosexuality. Two studies examined the coming out processes of gay grandfathers to their grandchildren. The results indicate that adult children were viewed as being a positive source during the coming out process. Grandfathers reported that coming out to their grandchildren was easier than coming out to their own children (Fruhauf et al., 2009; Orel and Fruhauf, 2013; Tornello and Patterson, 2016).

In terms of current equality policies, a broader diversity of gender and sexual identities who may experience different coming out processes should be taken into account. Here, the biographies and reactions of family members, such as pansexual and asexual people (in terms of sexual orientation) and transgender people and non-binary people (in terms of gender identity) provide new issues for future research.

### Practical Implications for Counseling

This study is a corner stone for a differentiated analysis of needs that different family members may have. Not all family members are equally affected by a coming out in a long-term heterosexual relationship and have different requests for possible support offers. To support as many family members as possible, if needed, the internet is the most relevant medium. In addition, counseling centers should provide support in multimedia form. Persons who made use of professional advice report positive experiences. However, those were consulted under high psychological and mental stress and were not easy to find. In general, advisory services should not only become more popular, their benefit should be presented more concretely, so that they can be used before the stress becomes too strong. Often families do not know which counseling service they can contact. It is vital that general providers indicate to be open for issues that are related with homosexuality and bisexuality, whereas LGBTQIA* counseling services indicate that also family members who feel affected by the coming out are welcome as clients. (LGBTQI+ is used as an abbreviation to describe people with different gender identities or sexual orientations including lesbians, gay men, bisexual people, trans*, queer, and intersex/-gender people. The plus symbolizes that the number of categories that are included in the abbreviation is not limited, it is open to additional identities.) It is important that advisory services communicate openness for these issues online and on-site.

When integrating the theoretical models on coming out processes and the empirical results, for the practical work it can be concluded that a coming out in a long-term heterosexual relationship is closely linked with the emotions and reactions in the family system. The finding that relationships with parents, children, and partners can improve as time passes suggests that they also process the coming out in stages, comparable to individuals coming out (Cass, 1979). Accepting these dynamics and integrating them into counseling can therefore be beneficial for the overall process for all involved.

The findings demonstrate that gender differences are an important aspect in the accessibility to advisory services. While heterosexual female partners have functioning online-platforms in German-speaking countries and built networks, there is a lack of support services men feel they can address. This suggests that more negative attitudes toward homosexuality should be regarded when counseling men (Whitley, 2001; Herek, 2002; Tucker and Potocky-Tripodi, 2006). Currently, counseling services are hard to find for persons who come out in a long-term heterosexual relationship and their family members. They all would benefit from a centralized opportunity to contact when seeking for support services.

## Conclusion

From a societal perspective, the present study revealed that a greater public awareness and broader education on coming out in long-term heterosexual relationships is essential. Coming out in long-term heterosexual relationships needs to be understood as a symptom of long-lasting heteronormative societal views about sexuality, gender, and family scripts (see Dieckmann and Steffens, 2014; Dieckmann, 2017). People either choose consciously to follow a heterosexual life although they knew about their homosexuality or bisexuality in order to fit into societal expectations, or their awareness of their non-heterosexual feelings arose later in life because of their heteronormative socialization. However, a greater acceptance in society for sexual diversity is needed to give people the opportunity to develop their sexual identity during adolescence. Thus, less heteronormativity, less rigid gender and family scripts within society, and more education about non-heterosexual orientations would reduce the number of coming outs in long-term heterosexual relationships. LGB+ youth in general would be encouraged to disclose their sexual identity at an earlier stage of their life and older adults would be encouraged to disclose “more easily” their coming out – and their families could better handle the coming out situation and reach out to them with an accepting attitude.

## Data Availability Statement

The raw data supporting the conclusions of this article will be made available by the authors, without undue reservation.

## Ethics Statement

The studies involving human participants were reviewed and approved by Ethics Committee Friedrich-Schiller-Universität Jena. The patients/participants provided their written informed consent to participate in this study.

## Author Contributions

JD and CN drafted the manuscript. MS, JD, and CN revised the manuscript. All authors approved the final version of the manuscript.

## Conflict of Interest

The authors declare that the research was conducted in the absence of any commercial or financial relationships that could be construed as a potential conflict of interest.

## Publisher’s Note

All claims expressed in this article are solely those of the authors and do not necessarily represent those of their affiliated organizations, or those of the publisher, the editors and the reviewers. Any product that may be evaluated in this article, or claim that may be made by its manufacturer, is not guaranteed or endorsed by the publisher.

## Footnotes

1 Note that percentages need to be interpreted with caution in all studies because random (or convenience) sampling of participants or obtaining representative samples is not possible.
